# Cellulolytic bacteria in the large intestine of mammals

**DOI:** 10.1080/19490976.2022.2031694

**Published:** 2022-02-20

**Authors:** Alicia Froidurot, Véronique Julliand

**Affiliations:** Université Bourgogne Franche–Comté, Institut Agro Dijon, PAM UMR A 02.102, Dijon, France

**Keywords:** *Fibrobacter*, *Ruminococcus*, CAZymes, plant cell wall, strain isolation

## Abstract

The utilization of dietary cellulose by resident bacteria in the large intestine of mammals, both herbivores and omnivores (including humans), has been a subject of interest since the nineteenth century. Cellulolytic bacteria are key participants in this breakdown process of cellulose, which is otherwise indigestible by the host. They critically contribute to host nutrition and health through the production of short-chain fatty acids, in addition to maintaining the balance of intestinal microbiota. Despite this key role, cellulolytic bacteria have not been well studied. In this review, we first retrace the history of the discovery of cellulolytic bacteria in the large intestine. We then focus on the current knowledge of cellulolytic bacteria isolated from the large intestine of various animal species and humans and discuss the methods used for isolating these bacteria. Moreover, we summarize the enzymes and the mechanisms involved in cellulose degradation. Finally, we present the contribution of these bacteria to the host.

## Introduction

**Figure 1.**
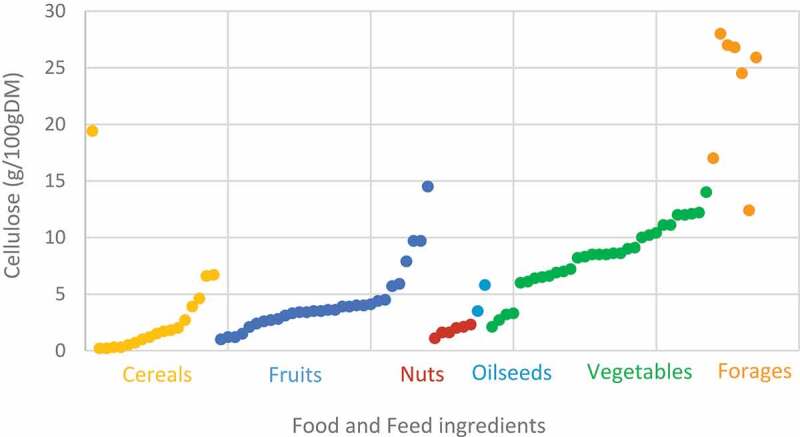
Percentage of cellulose measured in human and animal food.^17–19^

**Figure 2.**
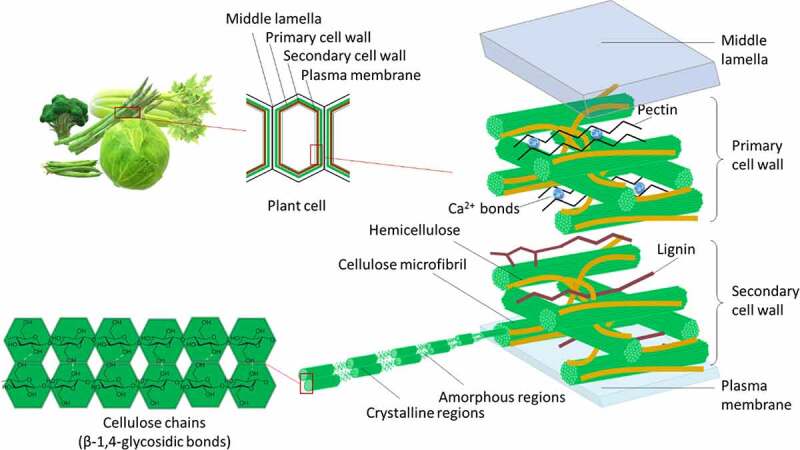
Schematic representation of the composition and structure of the plant cell wall and cellulose chain. In crystalline cellulose, chains are linked by hydrogen bonds in an orderly and periodic way, whereas in the amorphous cellulose regions, chains are disordered.

The participation of resident microbiota in the breakdown of dietary cellulose in the large intestine has long been recognized in both monogastric^1^ and ruminant^2,3^ herbivorous mammals. Similarly, in omnivorous mammals, including humans, the utilization of dietary cellulose by microorganisms inhabiting in the large intestine has also been a subject of interest since the nineteenth century (Weiske, 1870; von Tappeiner, 1883, Knieriem, 1885; Wicke, 1890; Barany, 1902 cited by Allen and Carlson 1927^4^). In 1906, Lohrisch reviewed the scientific knowledge of the time to underline the value of cellulose in the human organism.^5^ Investigation bloomed a century later in herbivorous^6–11^ and omnivorous^12–14^ mammals, triggered by the demonstration of the nutritional significance and beneficial effects of cellulose in gut health.^15,16^ Cellulose is a major component of plant cell walls, together with hemicellulose and pectin. The dry matter of the raw plant ingredients fed to herbivorous or omnivorous animals contains 10%–28% cellulose,^17^ and the percentage of cellulose in the dry matter of human foods can be up to 17%^18,19^ (Figure 1). Cellulose is a complex polysaccharide consisting of linked d-glucose units organized into either crystalline or amorphous cellulose (Figure 2). It is neither digested nor absorbed in the upper gut of mammals and is broken down only via a symbiotic association established between cellulolytic microorganisms and their host.^20–28^ Therefore, cellulolytic bacteria play a vital role in the valorization of energy from food and impact the host health. One of the major contributions of the cellulolytic microbiota is the provision of energy to the host via the metabolization of the complex chains of d-glucose units into short-chain fatty acids (SCFAs) in the large intestine of both omnivorous^29^ and herbivorous^30^ animals. SCFAs also have various implications in the maintenance of good health. SCFAs have beneficial effects on intestinal membrane integrity, local intestinal immunity and play a role in microbiota-gut-health communication. Consequently, there is an increased interest for dietary fiber.

**Figure 3.**
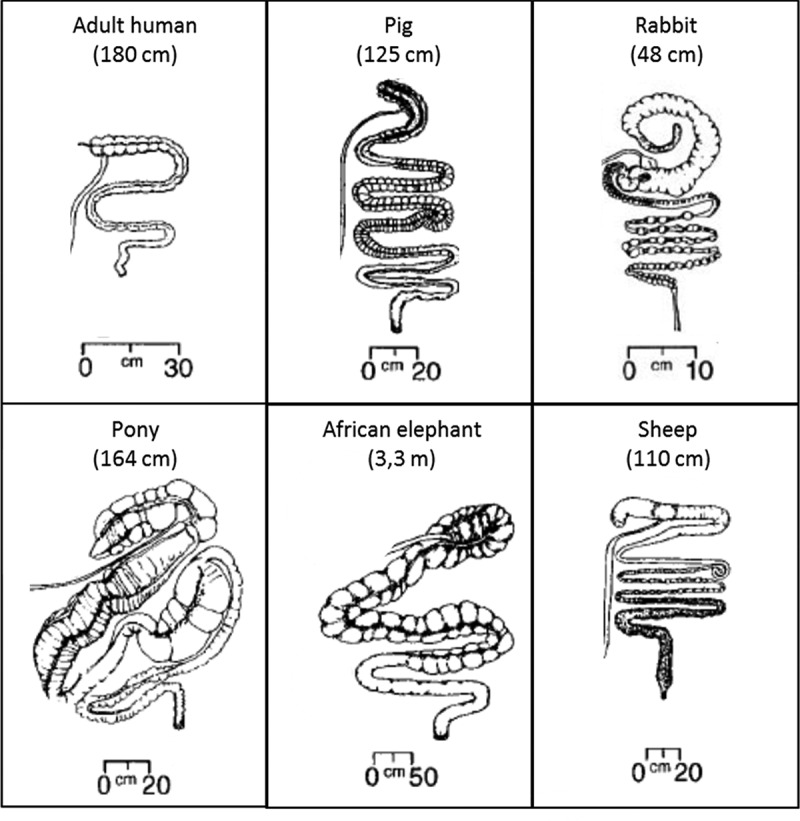
Large intestine of herbivorous and omnivorous mammals^31^. The body length is indicated in parentheses. Large intestine follows the small intestine and begins at the cecum and includes the appendix (humans only), colon, rectum, and anus.^32^

The large intestine of mammals (Figure 3) is a fermenter in which environmental conditions are favorable to microbial activity. It is the part of the digestive tract that follows the small intestine and begins at the cecum and includes the appendix (humans only), colon, rectum, and anus.^32^ The large intestine contains a minority of microorganisms that are able to degrade cellulose, including bacteria, and certain anaerobic eukaryotes (fungi and protozoa).^16,33^ In contrast, the abundance of microorganisms growing on soluble polysaccharides resulting from the “primary” cellulose degradation is high.^16,34^ In the present review, cellulolytic bacteria were focused. Despite their small quantity, cellulolytic bacteria play a crucial role, i.e., a “keystone” role, in this process, as their absence would, e.g., greatly decrease the degradation and utilization of an important substrate, thus affecting the remainder of the microbial community.^33^

After a brief retracing of the history of the discovery of cellulolytic bacteria in the large intestine, this review will present the current knowledge on the cellulolytic bacteria residing in the large intestine of mammals, including humans. Data applicable to the rumen are also presented for comparison or contrast. We will also focus on the isolation and identification of cellulolytic bacteria from the large intestine of various mammals, as well as on the enzymes and the mechanisms involved in the breakdown of cellulose. For each cellulolytic species described, we will first assess those that are detected by molecular biology techniques from the DNA of the microbiota of mammalian species, followed by those that have been isolated and cultured from samples of the large intestine microbiota of different mammals.

## History of the investigation of cellulolytic bacteria in the large intestine

**Figure 4.**
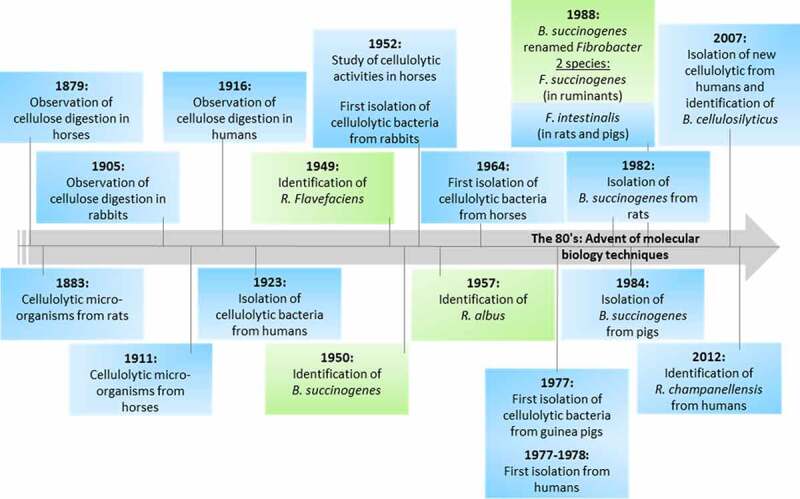
Brief summary of the time points of research published on cellulolytic bacteria observed and isolated from the rumen (green) and the large intestine (blue).

In his review published in 1946,^35^ Hungate stresses that the establishment of the fact of cellulose digestion was demonstrated as early as 1879 in the horse (Ellenberger, 1879 cited by Hungate, 1946^35^) and 1905 in the rabbit (Ustjanzew, 1905, cited by Hungate, 1946^35^). In humans, the first results showing that man is also able to digest the cell walls of vegetables and fruits date from 1916^36–39^ (Figure 4).

These observations were followed by the investigation of the microorganisms that are responsible for the breakdown of cellulose, after Von Tappeiner proved the symbiotic nature of the process via the demonstration of volatile acids, carbon dioxide, and methane as final products (von Tappeiner, 1883 cited by Hungate, 1946^35^). Pioneer works were conducted at the beginning of the 20^th^ century that showed the presence of microorganisms capable of utilizing cellulose in the digestive tract of cows^3^ and horses^1^. Khouvine (1923)^40^ was the first researcher who succeeded in isolating an anaerobic cellulose-splitting bacterium in a pure culture from the human intestine.^41^ This bacterium, named *Bacillus cellulosae dissolvens*, was capable of decomposing cellulose actively, with the formation of acids and gases (much less vigorously, however, in pure than in crude culture), and grew only in the presence of a fecal extract. Later, other cellulolytic bacteria were isolated from the large intestine of various animal species, including humans. This isolation work benefited from the original work of Hungate, who isolated strains of cellulolytic bacteria in 1946 in the cattle rumen using a new technique.^42^ In 1950, Hungate observed Gram-negative rods, identified as *Bacteroidetes succinogenes*, in the cattle rumen^43^ (later renamed *Fibrobacter succinogenes* by Montgomery^44^). Another rod, named “the less actively cellulolytic rumen rod” in 1946, was identified as *Butyrivibrio fibrisolvens* in 1956. The intestinal strains of cellulolytic bacteria were identified based on the observation of phenotypic characteristics, including those derived from morphological, biochemical, and physiological tests. Gram-positive cocci were found in the rabbit cecum.^45^ They resembled the *Ruminococcus flavefaciens* found in the rumen,^46^ but did not produce a yellow pigment and were probably closer to the *Ruminococcus albus* identified later in the rumen.^47^ Several cellulolytic bacteria were isolated in the horse large intestine: a Gram-negative rod resembling the one isolated from the rumen^43^ and classified in the genus *Bacteroides*, another Gram-negative rod, one Gram-negative spore former resembling *Bacillus cellulose dissolvens*, one Gram-negative coccobacillus, and a Gram-negative curved rod.^48^ Later, several strains that morphologically resembled *R. flavefaciens* were isolated from donkey and pony cecum.^20^ In the Guinea pig cecum, Gram-variable cellulolytic cocci were isolated, belonging to the genus *Ruminococcus* but different from those described previously in the rumen.^49^ In human feces, cellulolytic *Bacteroides* spp. different from the *Bacteroides succinogenes* isolated by Hungate, cellulolytic *Ruminococcus* spp., *Clostridium* spp., and *Eubacterium* were found.^50–53^ In the same period, strains from pig fecal samples^23^ and rat cecum^21,22^ were identified as *R. flavefaciens* and *B. succinogenes*.

The advent of molecular biology techniques has led to the reclassification of some cellulolytic bacteria species. In 1988, using comparative 16S rRNA sequencing of several strains of *B. succinogenes*, it appeared that these strains were not closely related to the other species of *Bacteroides*, but belonged to a new genus *Fibrobacter*. In addition, it revealed that the strains isolated from the rat and pig cecum belonged to a new species of the genus *Fibrobacter* called *F. intestinalis*.^44,54^ As for the genus *Ruminococcus*, it was divided into two phylogenetically separate groups using the 16S rRNA gene in 1995.^55^ Group I, including *R. flavefaciens*, the type species of the genus, was suggested as belonging to the family *Ruminococcaceae*, whereas several species of *Ruminococcus* were reclassified as *Lachnospiraceae*.^55,56^ Among the different species of *Ruminococcus* in the family *Ruminococcaceae*, three are cellulolytic: *R. flavefaciens, R. albus*, and *Ruminococcus champanellensis*.^57^ The family *Lachnospiraceae* does not contain cellulolytic species.

Over a century of research on the large intestinal cellulolytic microorganisms has brought to light two major cellulolytic bacterial genera, *Fibrobacter* and *Ruminococcus*, in herbivores as well as in omnivores, such as humans. These genera will be described further.

## Cellulolytic bacteria belonging to the genus *Fibrobacter*

Using molecular approaches, the genus *Fibrobacter* was detected in the intestinal tract of several herbivorous (*Equidae, Elephantidae*, and *Leporidae*) and omnivorous (*Muridae* and *Hominidae*) families. In pigs, the phylum *Fibrobacteres*, recently renamed *Fibrobacterota*,^58^ was detected. Currently, no strain of either *F. succinogenes* or *F. intestinalis* has been isolated or identified from the human large intestine.

In the horse cecum, *Fibrobacter* (particularly *F. succinogenes*) was detected by qualitative polymerase chain reaction (q-PCR) using oligonucleotide probes.^59,60^ In the pony and donkey cecum, *F. succinogenes* was identified,^20^ whereas no *F. intestinalis* was detected. Later studies suggested the presence of two new *Fibrobacter* lines in the pony, as there was no hybridization with the three existing *F. succinogenes* 16S rRNA-targeted oligonucleotide subspecies specific probes.^61^ It is possible that these lines were the new subgroups isolated recently: subgroups V and VI.^62^ In the elephant, *F. succinogenes* was also identified in feces by q-PCR.^63,64^ Both species, *F. succinogenes* and *F. intestinalis*, were identified in the rabbit cecum via dot-blot hybridization with 16S rRNA-targeted oligonucleotide probes.^65^ In omnivorous mammals, *F. intestinalis*, but not *F. succinogenes*, was detected in the mouse cecum by fluorescent-dye-conjugated oligonucleotides,^66^ whereas *Fibrobacter succinogenes* alone was identified in the wild gorilla feces using a 16S rRNA gene clone library and T-RFLP.^67^ Although culture-independent methods had shown the presence of *F. succinogenes* and *F. intestinalis* in large intestine-fermenting mammals, these strains were isolated only recently.^62^

Approximately 20 strains of *F. succinogenes* have been isolated exclusively in herbivorous mammals, either from the cow cecum^54,66^ or the various animals feces: horse, tapir, capybara, rhinoceros, colobus monkey, and elephant^62^ (Table 1). Conversely, the eight strains of *F. intestinalis* reported to date have been isolated in non-herbivorous mammals: the rat (from the cecum), rhesus monkey (from the feces), and pig (from the cecum or feces)^21,44,62^ (Table 1). In 2021, only the strain of *F. succinogenes* from the bovine cecum and four strains of *F. intestinalis* are in collection.

**Table 1.** Cellulolytic strains isolated from the large intestine and type strains isolated from the rumen: collection number, authors and date of isolation, metabolism and accession number for rRNA 16S sequences.^20–24,26–28,44,52–54,62,68–73^ The carbohydrates consumed and end products are indicated by the following letters: Glu, glucose; Cel, cellobiose; Mal, maltose; Lac, lactose; Ara, arabinose; Xyl, xylose; Gal, galactose; Fru, fructose; Sac, saccharose; Raf, raffinose; Rib, Ribose; Man, mannose; AG, galacturonic acid; Mel, melibiose; A, acetate; S, succinate; F, format; L, lactate; E, ethanol; H, hydrogen. ND, not determined

Cellulolytic species	Strains (collection number)	Isolated by	Date	From	Carbohydrates consumed	Major products	Accession number for rRNA 16S sequences	Ref
*F.succinogenes* subsp *succinogenes* (I)	S85 (ATCC 19169)	Bryant	1959	bovine rumen	Cel, Glu, Lac	S, A, F	AJ496032	^22,44,72,73^
*F.succinogenes* subsp *elongatus* (II)	GC5 (ATCC 51216)	Montgomery	1988	bovine cecum	Cel, Glu		M62688	^54^
	UW H4	Neumann	2017	horse feces	Cel, Glu	S, A, F	KY463346	^62^
	UW T2	Neumann	2017	tapir feces	Cel, Glu	S, A, F	KY463367	^62^
	UW P1	Neumann	2017	capybara feces	Cel, Glu	S, A, F	KY463355	^62^
*F.succinogenes* subsp *elongatus* (IV)	UW R2	Neumann	2017	rhinoceros feces	Cel, Glu	S, A	KY463358	^62^
	UW T3	Neumann	2017	tapir feces	Cel, Glu	S, A	KY463368	^62^
	UW CM	Neumann	2017	colobus monkey feces	Cel, Glu	S, A, F	KY463341	^62^
	UW R3	Neumann	2017	rhinoceros feces	Cel, Glu	S, A, F	KY463359	^62^
*F.succinogenes* (V)	UW H1	Neumann	2017	horse feces	Cel, Glu	S, A	KY463343	^62^
	UW H2	Neumann	2017	horse feces	Cel, Glu	S, A	KY463344	^62^
	UW H5	Neumann	2017	horse feces	Cel, Glu	S, A	KY463347	^62^
	UW H8	Neumann	2017	horse feces	Cel, Glu	S, A	KY463350	^62^
	UW T1	Neumann	2017	tapir feces	Cel, Glu	S, A	KY463366	^62^
	UW H9	Neumann	2017	horse feces	Cel, Glu, Lac	S, A	KY463351	^62^
	UW H3	Neumann	2017	horse feces	Cel, Glu	S, A	KY463345	^62^
	UW H6	Neumann	2017	horse feces	Cel, Glu	S, A	KY463348	^62^
	UW H7	Neumann	2017	horse feces	Cel, Glu	S, A, F	KY463349	^62^
*F.succinogenes* (VI)	UW EL	Neumann	2017	elephant feces	Cel, Glu	S, A	KY463342	^62^
	UW R1	Neumann	2017	rhinoceros feces	Cel, Glu	S, A	KY463357	^62^
	UW R4	Neumann	2017	rhinoceros feces	Cel, Glu		KY463360	^62^
*F.succinogenes* (VII)	UW P2	Neumann	2017	capybara feces	Cel, Glu	S, A	KY463356	^62^
*F.intestinalis* (I)	NR9 (ATCC 43854)	Montgomery	1982	rat cecum	Cel, Glu, Lac	S, A	AJ496284/M62695	^21,22,44^
	C1a	Varel	1984	pig cecum	Cel, Glu	S, A, E	M62686	^23^
	UW S1 (DSM 104696)	Neumann	2017	pig feces	Cel, Glu	S, A	KY463362	^62^
*F.intestinalis* (II)	DR7 (ATCC 43855)	Montgomery	1982	pig cecum	Cel, Glu	S,A	M62687	^44^
	UW S2 (DSM 104697)	Neumann	2017	pig cecum	Cel, Glu	S, A, F	KY463363	^62^
	UW S3	Neumann	2017	pig cecum	Cel, Glu	S, A, F	KY463364	^62^
*F.intestinalis* (III)	UW RM	Neumann	2017	rhesus monkey feces	Cel, Glu	S, A, F	KY463361	^62^
	UW S4	Neumann	2017	pig cecum	Cel, Glu	S, A, F	KY463365	^62^
*R.flavefaciens*	C94 (ATCC 19208, NCDO 2213)	Bryant	1958	bovine rumen	Cel	F, A, S, L	L76603/X83430	^69,70^
	FD1	Bryant	1958	bovine rumen	Cel	F, A, S, L	AF104844	^70^
	C52 (ATCC 49949)	Varel	1984	pig cecum	Cel, Ara	A, S, E		^23^
	BCL1	Macy	1982	rat cecum	Cel	S, A		^21^
	AA	Julliand	1996	donkey cecum	Cel, Glu, Xyl, Gal, Fru, Mal, Lac, Sac, Raf	A, L, E, S		^20^
	AB	Julliand	1996	donkey cecum	Cel, Glu, Xyl, Gal, Fru, Mal, Lac, Sac, Raf	A, F, E, L, S		^20^
	AC	Julliand	1996	donkey cecum	Cel, Glu, Xyl, Gal, Fru, Mal, Lac, Sac, Raf, Man, Ara	L, F, A, E, S		^20^
	AD	Julliand	1996	donkey cecum	Cel, Glu, Xyl, Gal, Fru, Mal, Lac, Sac, Raf, Man	L, E, A, F, S		^20^
	AE	Julliand	1996	donkey cecum	Cel, Glu, Xyl, Gal, Fru, Mal, Lac, Sac, Man	E, F, A, S, L,		^20^
	PA	Julliand	1996	pony cecum	Cel, Glu, Xyl, Gal, Fru, Mal, Lac, Sac	A, E, F, S		^20^
	PB	Julliand	1996	pony cecum	Cel, Glu, Xyl, Gal, Fru, Mal, Lac, Sac, Raf	F, A, E, S, L		^20^
	PC	Julliand	1996	pony cecum	Cel, Glu, Xyl, Gal, Fru, Mal, Lac, Sac, Raf, Man	L, F, A, E, S		^20^
*R.albus*	7 (ATCC 27210, DSM 20455, NCDO 2250)	Hungate	1957	bovine rumen	Cel, Glu, Sac, Lac, Man	F, E, A, H, S	L76598/X85098	^69,70^
*R.champanellensis*	18P13 (DSM 18848, JCM 17042)	Robert et al	2003	human feces	Cel	A, S, H, E, F, L	AJ515913	^28,68^
	25F7	Robert et al	2003	human feces	ND	ND		^28,68^
*Ruminococcus* spp	HS6	Montgomery	1988	human feces	ND	S, A		^52^
	HS3	Montgomery	1988	human feces	ND	S, A		^52^
	W8	Wedekind	1988	human feces	ND	S, A, F, L		^53^
	W11	Wedekind	1988	human feces	ND	S, A, F		^53^
	HS7	Montgomery	1988	human feces	ND	E, A, H, L, F		^52^
*B.cellulosilyticus*	CRE21 (DSM 14838, CCUG 44979)	Robert et al	2007	human feces	Glu, Sac, Fru, Mal, Xyl, Gal, Ri, Mel, Man, Lac, AG	A, P, S, H, L, F	AJ583243	^26^
	35AE31	Chassard et al	2010	human feces	ND	ND		^27^
	35AE37	Chassard et al	2010	human feces	ND	ND		^27^
	35AE34	Chassard et al	2010	human feces	ND	ND		^27^
	35AE35	Chassard et al	2010	human feces	ND	ND		^27^
	31S15	Chassard et al	2010	human feces	ND	ND		^27^
	31S18	Chassard et al	2010	human feces	ND	ND		^27^
*Enterococcus* spp	7L76	Robert et al	2003	human feces	ND	A, S, H, E		^68^
	27C63	Robert et al	2003	human feces	ND	ND		^68^
	18P16	Robert et al	2003	human feces	ND	ND		^68^
	7SE20	Chassard et al	2010	human feces	ND	A, S, H		^27^
	8SE23	Chassard et al	2010	human feces	ND	A, S, H		^27^
	8SE26	Chassard et al	2010	human feces	ND	A, S, H		^27^
*Eubacterium* spp	HS2	Montgomery	1988	human feces	ND	A, E		^52^
*Clostriduim* spp	W10	Wedekind	1988	human feces	ND	E, A, F, L, H		^53^
*Clostridium herbivorans*	54408	Varel	1992	pig feces	Cel, Mal	F, B, H, E		^24,71^

The common traits of the 29 strains of *F. succinogenes* and *F. intestinalis* isolated to date include the ability to consume the carbohydrates released by the cellulose degradation, glucose, and cellobiose utilization, and the inability to grow on xylane, although they possess the enzymes to degrade it, and to ferment pentoses. Another characteristic of all members of *Fibrobacter* is the production of succinate as the major end-fermentation product of cellulose, as well as acetate (in lesser amounts).

*F. succinogenes* are Gram-negative nonmotile, nonsporulating pleomorphic cells (rods or ovoid). Their DNA G + C content is 48%–49%. Some strains can growth on lactose, in addition to glucose and cellobiose, and some strains isolated from the large intestine (clade C, described below) can use urea as a source of nitrogen.^74^ Some strains also produce formate in addition to succinate and acetate.^62^

*F. intestinalis* are Gram-negative nonmotile and nonsporulating rod cells. Some strains can metabolize maltose in addition to glucose and cellobiose. All strains produce succinate and acetate, and some can produce formate and ethanol in small quantities.^22,23,62^ Their DNA G + C content is 45%.^44^

**Figure 5.**
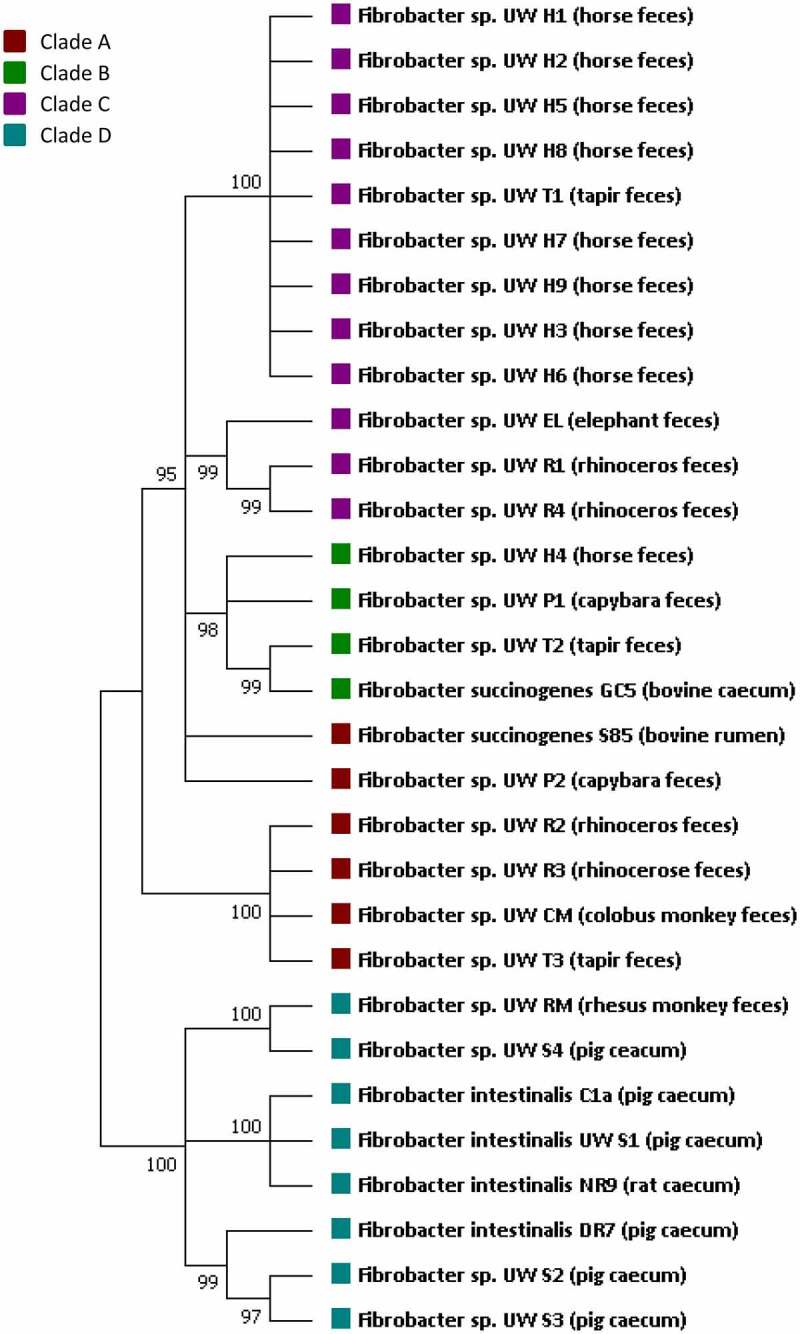
Molecular phylogenetic analysis of cellulolytic *Fibrobacter* found in the large intestine and of the type strain S85 using a maximum likelihood algorithm method (1,000 bootstrap trial). The numbers indicated for each branch represent bootstrap values. The phylogeny tree was constructed using a near-full-length 16S ribosomal RNA sequence (1262 bp) with MEGA 7.0 to the same length for all sequences. The subgroups of *F. succinogenes* were classified into three clades: subgroups I, IV, and VII in clade A; subgroup II in clade B; and subgroups V and VI in clade C.^74^ Three different lineages of *F. intestinalis* were identified after selective isolation and 16S rRNA sequencing.^62^ Subgroups I, II, and III of *F. intestinalis* were classified into clade D of *Fibrobacter*. Therefore, strain GH5, which was previously detected and classified into subgroup II, is now in clade B, whereas all others strains of *F. intestinalis* were classified into Clade D.

The genus *Fibrobacter* belongs to the family *Fibrobacteraceae* and phylum *Fibrobacterota*.^58^ The phylogenetic tree based on the 16S rRNA genes of the 29 strains of *Fibrobacter* spp. isolated from the large intestine is presented in Figure 5. We included in the tree *F. succinogenes* S85, originating from ruminants, and *F. intestinalis* NR9, from the rat cecum, which are the type strains for each species. The animal sources of each strain, their classification, and their metabolic characteristics are described in Table 1. Strains from bovine and ovine rumens were all grouped in subgroups I (formerly named subspecies *F. succinogenes*) and III (formerly named subspecies *F. elongatus* with other strains of subgroups II and IV),^54,62^ whereas strains from the large intestine of several herbivorous animals were only found in subgroups V and VI. The majority of strains from horses were included in subgroup V, whereas subgroups II and IV contained strains of both sources.^54,62^ All *F. intestinalis* strains were isolated from omnivorous animals, with the majority derived from the cecum. One strain isolated from pig feces (subgroup I) seemed to differ from the other strains isolated from the pig cecum (two in subgroup II and one in subgroup III). The other strain from feces was isolated from the rhesus monkey, which feeds on plants and insects. The phylogeny and taxonomy of the different strains of genus *Fibrobacter* are consistent with the observation that among other factors, diet is recognized as one of the main drivers responsible for shaping the genetic repertoire of the mammalian intestinal ecosystem.^75^

## Cellulolytic bacteria belonging to the genus *Ruminococcus*

Similar to the genus *Fibrobacter*, the genus *Ruminococcus* was detected in the intestinal tract of several herbivorous (*Equidae, Elephantidae*, and *Leporidae*) and omnivorous (*Muridae, Suidae*, and *Hominidae*) families.

*R. flavefaciens* was identified as the predominant cellulolytic bacterial species of the pony and donkey cecum using specific oligonucleotide probes. *R. albus* was also detected using the same method, albeit in smaller quantities.^20^ In another study, *R. flavefaciens* was the only cellulolytic *Ruminococcus* identified in the horse large intestine by molecular analysis of cloned 16S rRNA genes.^76^ *R. flavefaciens* and *R. albus* were predominant in the cecal content of conventional rabbits and specific pathogen-free rabbits.^65^ *R. flavefaciens* was also detected in elephants by q-PCR,^63,64^ and in monkey (rhesus) feces, but not in mouse, rat, or human feces using PCR detection.^77^ *R. flavefaciens* was also detected in gorilla feces by T-RFUP.^67^ In contrast, *R. albus* was detected in mouse, rat, monkey (rhesus), and human feces by PCR detection.^77,78^ Recently, using metagenome-assembled genomes (MAGs) in pig feces, MAG 0079 was found to represent the genome derived from the uncultivated strain of *R. flavefaciens*.^79^

Although *R. albus* was identified using molecular biology techniques, no strain has been isolated from the large intestine to date. One review mentions the isolation of *R. albus* from the pig, but no result was published.^80^ Ten strains of *R. flavefaciens* have been isolated, of which only two were from omnivores (pig and rat),^23,53^ whereas the majority was from herbivores (donkey and pony).^20^ Five strains of *Ruminococcus* spp. were isolated from human fecal samples.^52,53^ Two strains appeared to differ from previously described cellulolytic *Ruminococcus* strains, one strain formed end-products like those of *R. albus*, and two other strains resembled *R. flavefaciens* in fermentation products and chain formation by cells. More recently, several cellulolytic strains of *Ruminococcus* were isolated from human feces, with one being identified as *R. champanellensis*.^25,28,68^

Most strains isolated more than 20 years ago, such as the *Ruminococcus* spp. isolated from human feces by Montgomery (1988)^52^ or Wedekind (1988)^53^ or those isolated by Macy (1982)^21^ from the rat cecum and by Julliand (1999)^20^ from the pony and donkey cecum, are not available in culture collection. Only two cellulolytic strains of the genus *Ruminococcus* isolated from the large intestine were deposited in collection: one strain of *R. flavefaciens* isolated from the pig cecum (strain C52; ATCC 49949) and, the type strain 18P13 of *R. champanellensis* isolated from the human feces (DSM 18848, JCM 17042).

*Ruminococcus* are Gram-positive, nonsporulating, and nonmotile cocci. Three species degrades cellulose and metabolizes cellobiose. It also degrades xylane, but only some strains of the species can consume xylose.

The special features of *R. flavefaciens* include the production of a yellow pigment and the formation of chains by the cells. Some strains can consume xylose or other sugars, such as glucose, saccharose, fructose, lactose, or arabinose. *R. flavefaciens* produces formate, acetate, and succinate. The production of lactate and ethanol by this species is strain dependent and its DNA G + C content is 39%–44%. *R. albus* consumes xylose, glucose, saccharose, lactose, mannose, and fructose, depending on the strain. This species produces formate, ethanol, and acetate, and some strains can produce lactate, succinate, and dihydrogen in small quantities. Its DNA content is 42.6%–45.8%. *R. champanellensis* ferments cellulose and xylane and metabolizes cellobiose to acetate, succinate, dihydrogen, ethanol, and small quantities of formate and lactate. Its DNA G + C content is 53.07%. The type strain of *R. champanellensis* from human feces is 18P13, which is the only strain studied to date.

**Figure 6.**
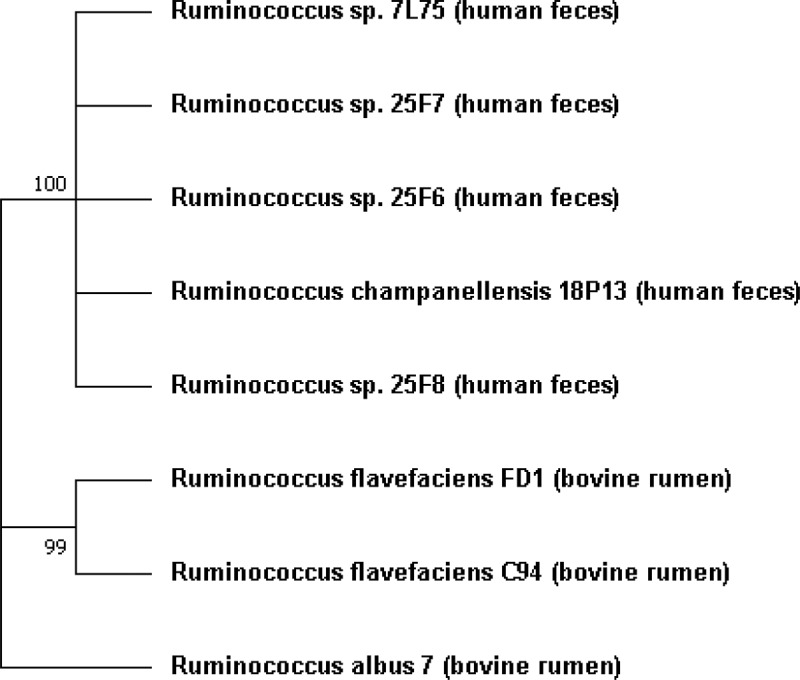
Molecular phylogenetic analysis of cellulolytic *Ruminococcus* found in the large intestine, the type strains *R. albus* 7 and *R. flavefaciens* C94, and the well-known strain FD1 using a maximum likelihood algorithm method (1,000 bootstrap trial). The numbers indicated for each branch represent bootstrap values. The phylogeny tree was constructed using a near-full-length 16S ribosomal RNA sequence (1315 bp) with MEGA 7.0 to the same length for all sequences.

*Ruminococcus*, from the family *Ruminococcaceae*, is a genus of *Clostridia* class bacteria.^69^ The phylogenetic tree based on 16S rRNA genes of the five strains of *Ruminococcus* spp. isolated recently from the human feces is presented in Figure 6 using the type strains *R. albus* 7 and *R. flavefaciens* C94 and FD-1. *R. flavefaciens*, C94, which was isolated by Bryant (1958)^70^ from the bovine rumen, has a lower cellulolytic activity than does strain FD-1, which was also isolated from the rumen and was studied more extensively. *R. flavefaciens* and *R. albus* were isolated from both herbivorous (horse and rat) and omnivorous (pig) animals whereas *R. champanellensis* strains were isolated exclusively from humans. Despite omnivorous diets different species of *Ruminococcus* were found in humans and pigs. In pigs, the same species as in herbivorous mammals were identified.

In fact, only one strain of *R. champanellensis* and one strain of *R. flavefaciens* were studied, and no strain of *R. albus* is currently available in culture collection to characterize species from the large intestine.

## Other cellulolytic bacterial genera isolated from the large intestine

Some cellulolytic strains of the *Bacteroides, Enterococcus, Eubacterium*, and *Clostridium* genera were isolated from the large intestine of omnivorous mammals (Table 1); however, the studies of these strains have not been completed. Among these strains, only the strain of *Bacteroides* spp. is in collection.

Strains of the phylum *Bacteroidota* were isolated from human fecal samples.^26,50,51^ Robert and al. (2007)^26^ identified one strain as a new *Bacteroides* species, *Bacteroides cellulosilyticus*, and the type strain CRE21T is in collection (DSM 14838, CCUG 44979). The cells were Gram-negative, nonmotile, and nonsporulating rods. This newly identified cellulolytic bacterium grew on cellulose (Avicel, Sigmacell, and spinach cell wall) and exhibited poor growth on xylane. It consumed a great variety of sugars: glucose, saccharose, fructose, maltose, xylose, galactose, ribose, melibiose, mannose, lactulose, and galacturonic acid; and produced acetate, propionate, succinate, H_2_, lactate, and formate. Its DNA G + C content is 41.10%.

A Gram-positive coccus belonging to the genus *Enterococcus*, because it was close to *Enterococcus faecalis*, was isolated from a human fecal sample.^25,27,68^ Only one strain, 7L76, was characterized and was shown to produce mainly acetate, succinate, ethanol, and hydrogen.

A strain of *Eubacterium* spp. was isolated from human feces using pebble-milled filter paper cellulose. The strain was a motile rod that produced mainly acetate and ethanol, as well as lactate, formate, and hydrogen in smaller amounts.^52^

A cellulolytic strain of *Clostridium* spp. was isolated from human fecal samples. This was a spore-forming rod that produced ethanol, acetate, succinate, formate, d-lactate, and hydrogen.^53^ A sporulating rod was also isolated from the pig intestinal tract and identified as *Clostridium herbivorans*. It consumed cellulose (Whatman filter paper ball milled with flint pebbles for 18 h), cellobiose, glycogen, maltose, and starch, and produced formate, butyrate, and a low quantity of hydrogen and ethanol.^24,71^

These other cellulolytic species, which are much less characterized than *Ruminococcus* and *Fibrobacter*, have only been isolated from the large intestine of omnivores, such as pigs or humans, whereas the cellulolytic capacity of the rumen or the large intestine of monogastric herbivores has been studied more extensively. It can be hypothesized that other cellulolytic species unknown to date are present and will be further investigated.

## Methods for the isolation of cellulolytic bacteria

**Figure 7.**
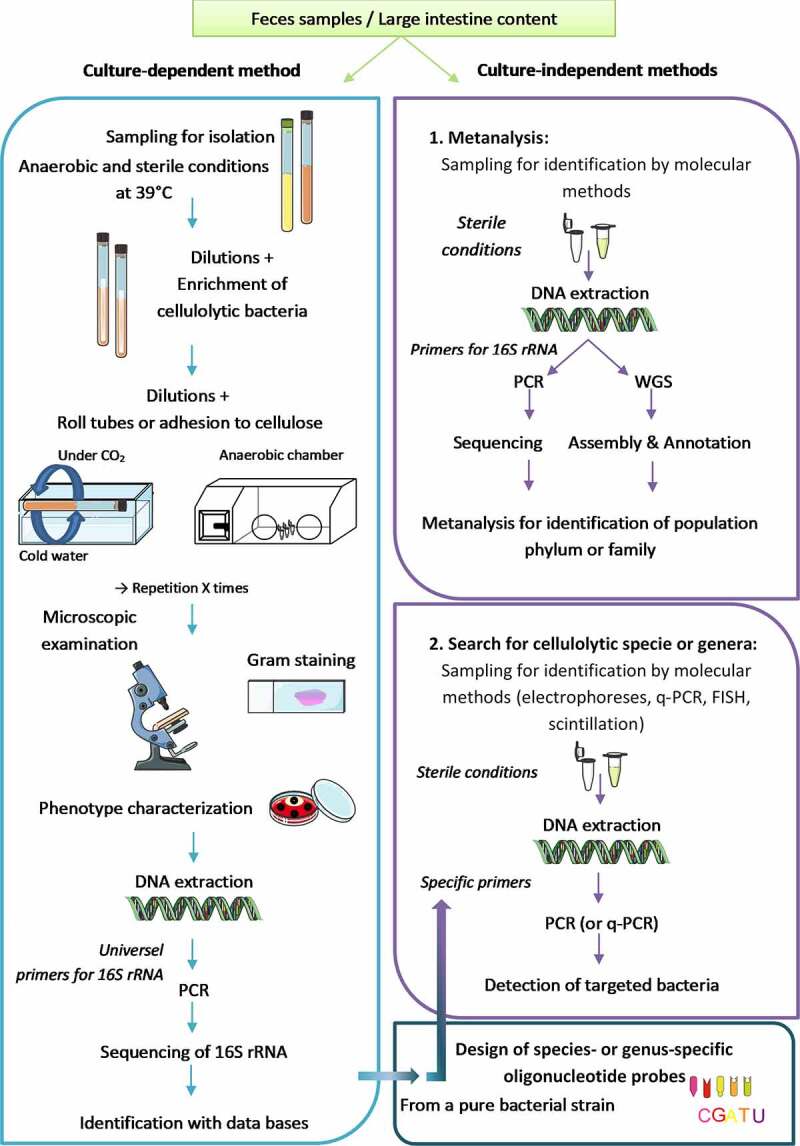
Culture-dependant methods used to isolate and further identify cellulolytic bacteria are described on the left. Culture-independent methods used to detect cellulolytic bacteria in the large intestine are described on the right. Two different techniques are employed to obtain pure cultures of cellulolytic bacteria: roll tubes and adhesion to cellulose. Phenotypic (colony shape, cell shape, Gram, nutrition, end products, …) or/and genetic approaches (16S rRNA sequencing, %GC, WGS) can be used to identified cellulolytic bacteria. Metagenomics or by specific probes are used in the detection of cellulolytic bacteria in intestinal contents. WSG: Whole sequence genome; q-PCR: real-time PCR; FISH: Fluorescence In Situ Hybridization.

Despite their crucial role, cultured representatives of cellulolytic bacteria living in the large intestine of mammals are lacking, resulting in insufficient functional characterization. Their specific growth requirements of strict anaerobic conditions on an insoluble substrate impose technical constraints. The methods used for the study of cellulolytic bacteria in the large intestine are shown in Figure 7.

The work of Khouvine (1923)^40^ represented the first successful method for isolating a microorganism from the intestinal flora of man which, when grown on cellulose caused it to be broken down and dissolved.^4^ The methods used by Kaar Sijpesteijn and Hungate, who were the first researchers to isolate cellulolytic strains, were similar. Hungate used cellulose prepared by treating cotton with hydrochloric acid, which was then diluted, filtered, washed, dried, and ground in a pebble mill for 72 h. Kaar Sijpesteijn used another culture medium using a strip of Whatman no. 1 filter paper as source of cellulose. In 1953, a medium to cultivate anaerobe bacteria of the rumen was using rumen fluid, glucose, cellobiose, and agar (RGCA medium), which allowed the isolation of cellulolytic bacterial strains without a source of cellulose.^81^ In all three cases, the method used for the culture and isolation of anaerobic bacteria was the roll tube method. This method comprises cooling tubes containing agar medium and the bacterial inoculum that are turned rapidly in cold water to give an even dispersion of substrate and inoculum in the agar. In this way, the agar medium is distributed as a thin layer over the interface surface of the tubes. This method is carried out under oxygen-free gas, preferably carbon dioxide, to displace the air and avoid the contact of oxygen with bacteria. The media were also prepared under oxygen-free gas. The presence of a reducing agent, such as sodium thioglycolate, cysteine hydrochloride, or sodium sulfite, is also very important to the culture of anaerobic bacteria. A colored indicator, such as resazurin, is often used to detect traces of oxygen by the increased redox potential. The roll tube method reported by Hungate has undergone many modifications and improvements^43,82,83^ since the first cellulolytic bacteria isolation.^42^ It was later used by many authors to isolate cellulolytic bacteria, first from the bovine and ovine rumen,^84–86^ and the medium and source of cellulose were adapted to the ecology of the large intestine of specific animal species. For example, to isolate cellulolytic bacteria from the rat cecum or from the ponie and donkey cecum, rat intestinal content and equine cecal content were used alone or mixed with rumen fluid, respectively.^20–22^ Pure cellulose or spinach cell wall were used to isolate cellulolytic bacteria from human feces.^26,28,52,53,68^

Recently, a new method was developed to select cellulolytic bacterial species of the genus *Fibrobacter* from many animals.^62^ This method consists in isolating bacteria adhering to cellulose powder (Sigmacell 50). A total of 45 axenic cultures of *Fibrobacter* were obtained from ruminants and monogastric herbivorous animals. Based on the analyses of 16S rRNA extracted from equine feces in the same study, many of the *Fibrobacter* species were shown to belong to phylotype VI, although no strains were isolated and 50% remain unclassified. The authors explained that the media formulation, which was based on a composition originally used for rumen bacteria, was a possible explanation for this observation, as it would lack specific growth factors and provide insufficient nutrition to stimulate growth. In addition, they did not use rumen or cecal fluid, as in previous works, for the isolation of cellulolytic bacteria.^20–23,26,28,50,52,53,68^ Another possibility considered by the authors is that bacterial populations not isolated but identified by culture-independent methods do not degrade crystalline cellulose.

After isolation, bacterial strains are identified by phenotypic characterization, including morphological, biochemical, and physiological tests according to Bergey’s manual. The cultural method is complemented by biological molecular techniques, such as the determination of the G + C content, 16S rRNA gene sequencing, and DNA homology, to identified isolated bacteria. The comparison of all of these characteristics with those of pure strains allows the identification of the newly isolated strains. Therefore, it is necessary to culture cellulolytic bacteria and to know how to isolate them. Few methods have been developed for the isolation of cellulolytic bacteria. However, the use of new techniques or new media could allow the discovery of new species. Fluorescent antibody staining technique has also been used on the past to investigate different bacterial strains isolated from horse cecum and colon.^88^ In addition, from a pure culture, it is possible to design nucleotide probes to detect a bacterium of interest in a complex ecosystem after a PCR or q-PCR by electrophorese or FISH method with fluorescent oligonucleotide probes.^20,63,64,66,67,74,79,87,88,89^ This technique allows targeting a genus or species in a more precise way compared with metagenomic analyses based on the 16S rRNA sequences of a bacterial population (Figure 7).

## CAZymes involved in cellulose degradation

Recently, several reviews have summarized the current knowledge on the enzymes involved in dietary fiber degradation, in particular cellulose degradation, notably in the gut microbiota.^16,90–92^ The enzymes responsible for carbohydrate degradation, modification, or creation are commonly named carbohydrate-active enzymes (CAZymes) and are indexed in the CAZy database (http://www.cazy.org/).^93^ This database classifies proteins into 6 families based on their mode of action: glycoside hydrolases (GHs), carbohydrate esterases, polysaccharide lyases, glycosyltransferases, and auxiliary activities. In turn, carbohydrate-binding modules (CBMs) are noncatalytic modules of CAZymes that help target enzymes to their substrates. The classification of CAZyme families is defined based on the significant similarity of amino acid sequences that do not necessarily correspond to one function. The presence of enzymes that act on different substrates within the same family is also possible.^93^

**Figure 8.**
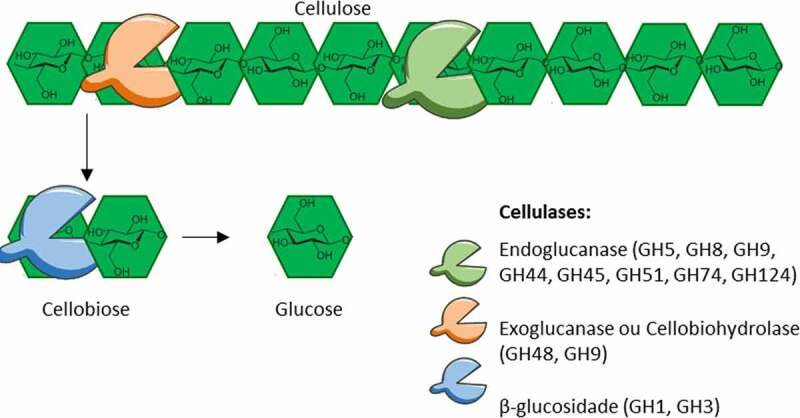
Schematic representation of cellulose degradation by the three enzyme families found in cellulolytic bacteria isolated from the large intestine and in the rumen for the type strains. Endoglucanase access long molecules of cellulose and cleave at a random position in the chain; exoglucanases or cellobiohydrolases cleave cellodextrins of defined sizes at the nonreducing ends and release cellobiose; and β-glucosidases hydrolyze cellobiose or cellulo-oligomer (up to 6 molecules of glucose).

Initially, this classification concerned cellulases, the enzymes that degrade cellulose.^94^ CAZymes are often multimodular, as they can contain several domains of different families. Cellulases are GHs that cut the β-1,4-d-glucose often associated with one or more CBMs, with the CBM being a noncatalytic protein or internal peptide attached to the catalytic domain. According to the CAZy database, cellulases are found in at least 16 GH families (GH5–9, GH12, GH44–45, GH48, GH51, GH74, and GH124). Cellulases are classified according to their mode of action (Figure 8): endoglucanases (EC 3.2.1.4), which access long molecules of cellulose and cleave at a random position in the chain; exoglucanases or cellobiohydrolases (EC 3.2.1.176), which cleave cellodextrins of defined sizes at the nonreducing ends and release cellobiose; and β-glucosidases (EC 3.2.1.21), which hydrolyze cellobiose or cellulo-oligomer (up to 6 molecules of glucose).^90^ These different types of cellulases work in synergy to completely hydrolyze crystalline cellulose. Few genes in the genomes of humans or other animals encode CAZymes. In the human genomes, 97 GHs were found, and the substrates used by these enzymes are starch, maltose from starch, isomaltose, lactose, sucrose, and trehalose.^95^ Although an endoglucanase-like protein of the GH9 family was identified, its substrate remains unknown.

### From isolated bacterial strains

In the large intestine, the genes encoding cellulases found in the genome of isolated bacteria belong to the GH5, GH8, GH9, GH44, GH45, GH48, GH51, and GH74 families of endoglucanases, GH48 and GH9 families of cellobiohydrolases (Table 2), and GH1 and GH3 families of β-glucosidases.^16,74^

**Table 2.** Denomination and numbers of cellulases encoded by the genome of cellulolytic bacteria stains isolated from the large intestine and of type strains isolated from the rumen.^16,74,96–101.^

					Cellulases										Total GHs	Total CAZymes	Ref.
Ecosystem		Bacterium	Strains	Collection N.	GH5	GH8	GH9	GH44	GH45	GH48	GH51	GH74	GH124	Total			
Large intestine	human colon	*Ruminococcus champenellensis*	18P13	DSM 18848, JCM 17042	6	1	6	1		1		1		16	56	87	^97^
	rhesus monkey feces	*Fibrobacter succinogenes*	UW_RM		10	3	5	1	2		1			22	85.1 ± 17.3*	190 ± 30.4*	^74^
	colobus monkey feces		UW_CM		12	4	8	1	3		1			29	85.1 ± 17.3*	190 ± 30.4*	^74^
	horse feces		UW_H5		14	4	7	1	2		1			29	85.1 ± 17.3*	190 ± 30.4*	^74^
			UW_H1		14	4	6	1	2		1			28	85.1 ± 17.3*	190 ± 30.4*	^74^
			UW_H9		14	4	6	1	2		1			28	85.1 ± 17.3*	190 ± 30.4*	^74^
			UW_H6		14	4	6	1	2		1			28	85.1 ± 17.3*	190 ± 30.4*	^74^
			UW_H3		14	4	6	1	2		1			28	85.1 ± 17.3*	190 ± 30.4*	^74^
			UW_H8		14	4	6	1	2		1			28	85.1 ± 17.3*	190 ± 30.4*	^74^
			UW_H4		11	2	7		2		1			23	85.1 ± 17.3*	190 ± 30.4*	^74^
	tapir feces		UW_T1		12	4	6	1	2		1			26	85.1 ± 17.3*	190 ± 30.4*	^74^
			UW_T3		13	4	8	1	3		1			30	85.1 ± 17.3*	190 ± 30.4*	^74^
			UW_T2		14	6	10	1	6		1			38	85.1 ± 17.3*	219	^74^
	elephant feces		UW_EL		16	4	7	0	3		1			31	85.1 ± 17.3*	190 ± 30.4*	^74^
	rhinoceros feces		UW_R4		14	5	7	1	3		1			31	85.1 ± 17.3*	190 ± 30.4*	^74^
			UW_R1		14	5	7	1	3		1			31	85.1 ± 17.3*	190 ± 30.4*	^74^
			UW_R3		12	4	9	1	3		1			30	85.1 ± 17.3*	190 ± 30.4*	^74^
			UW_R2		13	6	8	1	4		1			33	85.1 ± 17.3*	190 ± 30.4*	^74^
	capybara feces		UW_P2		15	5	7	1	5		1			34	85.1 ± 17.3*	190 ± 30.4*	^74^
	pig ceacum	*Fibrobacter intestinalis*	UW_S3		10	5	5	1	2		1			24	85.1 ± 17.3*	190 ± 30.4*	^74^
			UW_S2	DSM 104697	10	5	5	1	2		1			24	85.1 ± 17.3*	190 ± 30.4*	^74^
			UW_S1	DSM 104696	9	3	4	1	2		1			20	85.1 ± 17.3*	190 ± 30.4*	^74^
			UW_S4		10	3	5	1	2		1			22	85.1 ± 17.3*	190 ± 30.4*	^74^
	rat ceacum		NR9	ATCC 43854	9	3	5	1	2		1			21	85.1 ± 17.3*	190 ± 30.4*	^74^
	porc ceacum		DR7	ATCC 43855	4	2	4		1		1	1		12	ND	ND	^98^
Rumen		*Fibrobacter succinogenes*	S85	ATCC 19169	10	5	7	1	3		1			27	103	186	^74^
		*Ruminococcus flavefaciens*	FD-1		14		12	1		1				28	101	140+	^16,99,100^
		*Ruminococcus albus*	7	ATCC 27210, DSM 20455	13	1	8	1		1		2	1	24	99	148	^96,101^

*a range was given by the authors

Regarding the genus *Fibrobacter*, all strains isolated and studied by Neumann et al. (2017)^62^ contained genes for cellulase in the GH5, GH8, GH9, GH44, GH45, and GH51 families.^74^ A greater number of CAZymes involved in plant-cell-wall polysaccharide degradation were found in *F. succinogenes* compared with *F. intestinalis*.^74^ *R. champanellensis* isolated from the human feces is the only *Ruminococcus* representative living in the large intestine with a sequenced genome. Genes for cellulase in the GH5, GH8, GH9, GH44, GH48, and GH74 families were identified. These bacteria exhibited fewer genes encoding GHs and cellulases compared with the better-known *Ruminococcus* strains of the rumen (Table 2). *R. champanellensis*, which was isolated from human feces,^28^ is less used to degrade recalcitrant fibers than are ruminal bacteria. One cellulase in the GH124 family was identified in one *R. albus* strain isolated from the rumen;^96^ however, this enzyme has not been studied in the large intestine samples or *Ruminococcus* strains from the large intestine.

For both cellulolytic *Fibrobacter* and *Ruminococcus* genera, cellulases are mainly found in the GH5 and GH9 families. Interestingly, the *F. succinogenes* strains isolated from the large intestine of horses, tapir, elephant, and rhinoceros (clade C) had more cellulase of the GH5 family than did ruminal strains, suggesting a potential adaptation to their environment. All strains of *Ruminococcus* produce GH48 enzymes, which play a role in cellulose hydrolysis as cellobiohydrolases. In contrast, *Fibrobacter* lacked the GH48 exoglucanase sequence.^74^ Cellobiohydrolases exist in *F. succinogenes* as atypical members of the GH9 family.^102^

In conclusion, cellulases are found in several GH families. Some families containing cellulases are common to all major cellulolytic bacteria (GH5 and GH9), whereas most of them are specific to a bacterial genus or species, which renders these bacteria unique.

### From intestinal microbiota

CAZymes have also been investigated directly via the sequencing of the intestinal microbiota genome. A greater number of total CAZymes was found in the bovine rumen, between 3,828 and 27,755 CAZymes according to previous studies, vs. 11,038 from the adult elephant gut, which is another large herbivorous mammal. In the Tiberian pig feces, which are omnivorous mammals, 13,000 carbohydrate-degrading genes were identified.^79^ Among carnivorous animals, only 372 and 440 CAZymes were detected in the Iberian lynx and giant panda feces, respectively, even if the latter is a carnivore that feeds on bamboo. Unexpectedly, 84 GH families were detected from Asian elephant microbiota.^103^ This high diversity of GH families was surprising compared with that known in the cow, another herbivorous mammal, in which 35–60 GHs were detected in the bovine rumen.^104,105^ In the fecal samples of giant pandas, 44 GH were found,^106^ whereas 42 GHs were identified in the Iberian lynx fresh fecal samples, a carnivore.^107^

In one study, further investigation was conducted to identify the number of candidate enzymes potentially involved in cellulose degradation. This was determined by focusing on all genes encoding enzymes of families including β-glucosidases, cellulases, and cellobiosidases or cellodextrin phosphorylases; i.e., the GH1, GH3, GH5, GH6, GH8, GH9, GH44, GH45, GH48, GH51, GH74, and GH94 families.^103^ In the bovine rumen, between 1,017 and 5,670 putative cellulolytic enzymes were found, whereas 2,074 enzymes were detected in the adult Asian elephant. There were only 39 and 124 putative cellulolytic enzymes in the Iberian lynx and giant panda, respectively. These results coincide with the diets of the species studied. In fact, there may be more putative cellulases in herbivores, such as elephants and cows, as there is a higher percentage of cellulose in their diets compared with carnivorous animals; therefore, the latter have a lower number of potential cellulases. However, a study compared the fibrolytic activities from the gut microbial ecosystems of 11 herbivores, including several ruminants and the horse, elephant, and zebra, after the isolation of enzymes.^108^ The enzymes of horse and zebra feces were more active on substrates (CMC, crystalline cellulose, cellobiose, and xylane) than were those taken from the rumen. The authors hypothesized that because the digestion time is shorter in monogastric animals, large intestine-derived microorganisms are more efficient at extracting maximum nutrients before excretion in the feces. Another study explored the activity of cellobiosidase and showed that it was equivalent between the samples assayed from cow rumen samples and lynx feces.^107^ Thus, the number of GHs does not necessarily reflect the efficiency of the enzymatic activity.

Only 12 putative cellulase genes were identified in giant panda feces, including three enzymes of the GH5 family and two of the GH8 family.^106^ This could explain why cellulose digestibility is weak in this mammal, even if it feeds on bamboo.^109^ In the Iberian lynx, only two enzymes in the GH5 family and four enzymes in GH51 were detected.^107^ A study of dogs on a high-fiber diet (7.5% of beet pulp) compared with a control diet (lower-fiber diet) revealed a greater amount of GHs in the controlled-diet setting, which was counterintuitive to the authors’ hypothesis.^110^ Although the total sequence number was different between samples (of the high-fiber and the controlled diet), the percentage of each gene within its gene family was similar for each of them. Interestingly, in the case of the high-fiber diet, more GHs belonged to families know to comprise cellulolytic enzymes, such a GH5 (31 vs. 18), GH8 (3 vs. 2), GH9 (2 vs. 0), and GH51 (19 vs. 10).

Cellulolytic GH-family genes have been associated with their phylogenetic affiliation. In the giant panda feces, half of the genes predicted for cellulases were found in species belonging to the genus *Clostridium*. Among 13 OTUs close to *Clostridium*, seven were only found in the panda compared with other mammals.^106^ The predicted genes for cellulases were found to be associated mainly with an OTU belonging to the genus *Clostridium* and were found only in the panda. The hypothesis of a new cellulolytic species of the genus *Clostridium* specific to pandas can be formulated. In adult Asian elephants, the enzymes belonging to the GH5, GH8, and GH9 families are mainly related to *Bacteroidales*. There are also many enzymes related to *Clostridiales* and *Fibrobacterales*. In addition, GH45 enzymes are found only in *Fibrobacterales*. There were 2% of *Fibrobacteres* in the adult Asian elephant gut. Moreover, 8% of the GH5 enzymes, which are the most numerous cellulolytic GHs, would belong to *Fibrobacteres*. It seems that *Bacteroidetes* and *Fibrobacteres* play an important role in the cellulose degradation in elephants.^103^ In pig feces, genes encoding cellulases (GH5, GH8, GH9, GH44, and GH45) were mainly identified in the phylum *Fibrobacterota*.^79^ Bacteria of the *Fibrobacteraceae* family possessed 54 GHs per genome.^79^

From different studies, it has become clear that both the number of CAZymes and the diversity of GH families depend on the animal species and diet. There are more putative cellulolytic enzymes in herbivorous than carnivorous animals, which coincides with the content of cellulose in the diets of the former.

## Mechanisms of cellulose degradation by gut bacteria

Among the cellulose-degrading bacteria that have been isolated from the large intestine, *Fibrobacter* and *Ruminococcus* spp. have developed different strategies for fibrolytic enzyme systems. The cellulosome was the first organization of cellulases to be discovered and described in *Clostridium thermocellum*.^111^

### The cellulosome of *R. champanellensis*

**Figure 9.**
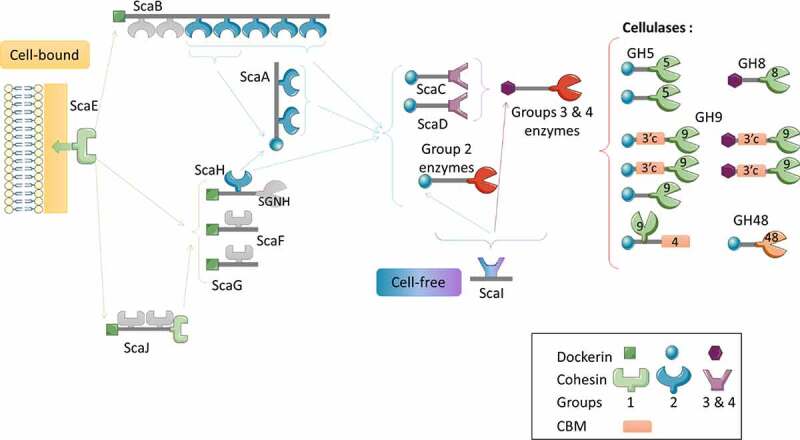
Schematic representation of cellulosome complexes in *R. champanellensis*^112^ and identified cellulases.^97^ The various types of cohesin–dockerin and cohesin–enzyme interactions are represented differently. The binding specificities of cohesin modules shown in light gray are undetermined. SGNH, lipase/esterase.

Noncatalytic elements of the cellulosome, i.e., the cohesin and dockerin modules, as well as scaffold-holding modules, were identified using sequencing analyses of the *R. champanellensis* genome.^112^ The cellulosome is a multienzyme complex that enables the bacterial cell to access and adhere to crystalline cellulose, which is then degraded by cellulases of the ultrastructure. Cohesin domains are found on the large scaffolding protein, which is the structural subunit. The interactions between the different subunits of the cellulosome are mediated by cohesin and dockerin modules. In addition to their catalytic domains, cellulases contain dockerin domains, which are anchored to cohesin domains.^113^ David et al. described the cellulosome of *R. champanellensis* (Figure 9). Two major types of cellulosome architecture were found: cell-bound and cell-free cellulosome systems. The entire cellulosome system contains 20 cohesin modules found on 12 scaffoldin proteins (ScaA to ScaL). The scaffoldins ScaA, ScaB, and ScaJ hold two, seven, and three cohesin modules, respectively, whereas the others have only one. Most of them have a dockerin module, which allows them to associate with the cohesins of other scaffoldins. ScaI has an unknow function but seems to possess a cell-free cellulosome architecture, unlike the other scaffoldins. Of the three types of cohesins currently known, two are found in the *R. champanellensis* cellulosome: CohC and CohD, as type I, are similar to CohC of *R. flavefaciens*. The others, which are also similar to *R. flavefaciens* cohesins, are classified as type III cohesin. There are 64 dockerins distributed into four groups. The dockerin–cohesin interactions of the group 1 allow cell anchoring with the ScaE scaffoldin. Most of the dockerin–cohesin interactions in group 2 are bound to GH enzymes, mainly cellulases or closely associated enzymes, and some contain a CBM module. Proteins bearing group 2 dockerin appear to play a major role in cellulose degradation. In groups 3 and 4, enzymes bearing dockerin are mostly hemicellulases; however, some dockerin-containing proteins lack confirmed carbohydrate-degrading components. Of the 107 CAZymes in the *R. champanellensis* genome, more than half are found on dockerin-containing proteins. Cellulases have been identified in families GH5, GH8, GH9, and GH48.^97^ All cellulases of families GH8, GH9, and GH48 have a dockerin module. Eight GH5 enzymes were found in the *R. champanellensis* genome. Three of them are cellulosomal, i.e., they interact with cohesins, and two of the three cellulosomal enzymes are cellulases. Four of the five remaining free enzymes of family GH5 are cellulases.^97^

The cellulosome systems of *R. flavefaciens* and *R. albus* isolated from the large intestine has not been described. It can be hypothesized that they have similar mechanisms as the ruminal strains. Similar to *R. champanellensis*, the ruminal strains of *R. flavefaciens* produce a particularly elaborate cellulosome encoding a large amount of dockerin and cohesin modules, including novel CBMs, and exhibit various combinations of dockerin-containing cellulases on their surface.^114,115^ These cellulosomes have been shown within several strains of *R. flavefaciens* using genome analyses demonstrating that the number of dockerins varies between 53 and 223.^116^ In contrast to *R. champanellensis* and *R. flavefaciens*, a lower abundance of dockerin molecules was found in the *R. albus* genome, suggesting a cellulosome;^117^ however, a single cohesin-containing protein was detected in two out of the three strains studied.^114^

### Fibro-slime proteins and outer membrane vesicles (OMVs) of *Fibrobacter*

The sequences of the scaffoldin and cohesin modules were missing in the genomes of *Fibrobacter* from the large intestine, as in the genome of the S85 type strain from the rumen.^74,118^ Another unusual fact of cellulolytic bacteria has been observed in *Fibrobacter*. Cellulases tended to occur without any identifiable associated CBMs.^74^ The unique copy of the gene encoding the GH51 endoglucanase also contained a CBM11 module. This gene has been identified in all *Fibrobacter* genomes from the large intestine.

The existence of OMVs containing CAZymes and a fibro-slime complex has been demonstrated in *F. succinogenes* S85, which is a reference strain isolated from the bovine rumen.^119^ Recently, a potentially cellulolytic multiprotein complex of degradative enzymes and fibro-slimes was identified. This complex, anchored to the outer membrane peptidoglycan, is thought facilitate the adhesion of *F. succinogenes* S85 to cellulose and subsequent cellulose degradation. The up-regulation of these proteins in cellulose-grown cells also indicates that the expression of the corresponding genes is controlled by catabolite repression. Cyclic di-guanidine monophosphate, known to regulate a variety of functions, has been proposed to be involved in cellulose degradation.^120^

Fibro-slime domains bring the substrate close to the cellulases located either in the outer membranes or coupled to extracellular secretion of endoglucanase.^121^ This is relevant to the fact that, in the same study, more CAZymes were found in the extracellular medium than in the periplasm or outer membrane. In addition, OMVs containing CAZymes are released from the bacterial cells to target plant cell walls. These OMVs contain fibro-slime proteins, cellulases, and hemicellulases. In addition to degrading cellulose, these vesicles had the capacity to degrade hemicelluloses and pectins, although *F. succinogenes* consumes only sugars released from cellulose degradation.^119^ Thus, OMVs would facilitate the access to cellulose in *F. succinogenes* S85 cells. To date, no search for OMVs in the large intestine strains of *Fibrobacter* spp. has been performed, but it is possible that some strains also use this system to reach cellulose and degrade it. In the genomes of various strains of *F. succinogenes* isolated from the large intestine of different mammals (horse, monkey, tapir, elephant, and capybara), proteins containing fibro-slime domains were identified. A smaller number of those proteins was observed in several strains of *F. intestinalis* isolated from omnivorous mammals.^74^ The lowest number predicted in a genome was 3 for *F. intestinalis*, whereas *F. succinogenes* strains had 8–10 distinct proteins containing a fibro-slime domain.^74^ The mechanisms of cellulose degradation by *F. succinogenes* and *F. intestinalis* would be a worthy focus in future studies, for describing better their role in cellulosic and hemicellulosic catabolism.

## Nutritional contribution of the large intestine cellulose degradation

**Figure 10.**
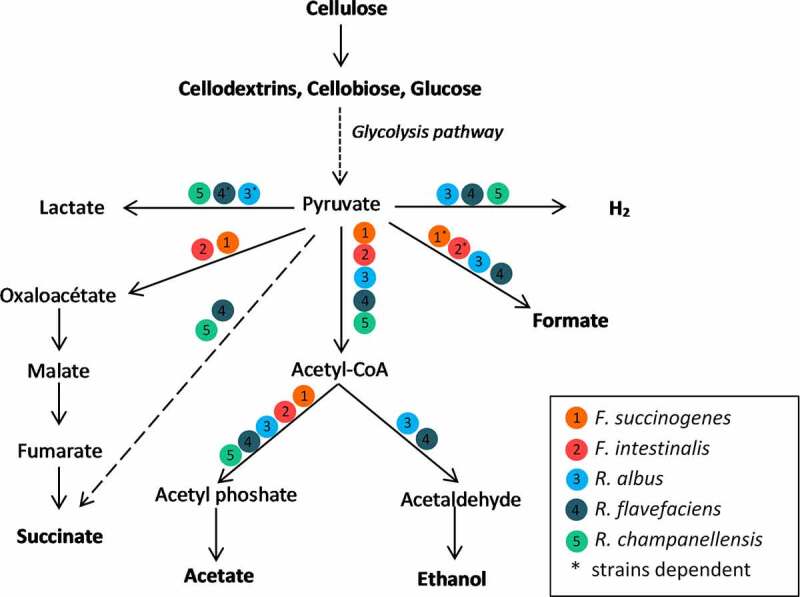
Metabolic pathways used by cellulolytic bacteria isolated from the large intestine and by type strains isolated from the rumen for the breakdown of cellulose. Each color and number correspond to a cellulolytic species. The end products and substrates are indicated in bold.

The microbial anaerobic breakdown and hydrolysis of cellulose in the large intestine of mammals result in the production of SCFAs, mainly acetate.^122^ The metabolic pathways used by major cellulolytic bacteria for the breakdown of cellulose are summarized in Figure 10.

The mean concentrations of SCFAs in the cecal contents of herbivorous (cattle, goat, sheep, donkey, horse, rabbit, and Guinea pig) and omnivorous (rat, hyrax, dog, and pig) mammals were summarized and reported to be near 100 mmo1∙1,^−1^ although variation exists.^122^ SCFAs contribute to supporting the host to achieve nutritional profit from the ingested plant cell walls.

In herbivorous mammals, the contribution of SCFAs to energy requirements is critical. For example, in the horse, which is a large large intestine-fermenting herbivorous animal, the microbial digestion of dietary cellulose can reach 40%,^123^ and the contribution of SCFAs has been estimated to be between 50% and 70%,^124,125^ with 30% originating from the cecum.^126^ In the rabbit, which is a smaller large intestine-fermenting herbivorous animal, it was also estimated that 30% of the energy requirements are derived from the SCFAs produced in the cecum, with cellulose digestion averaging 20%.^127^ In ruminants, the contribution of the large intestine to energy production remains poorly explored, in contrast to that of the rumen. However, some data emphasized the nutritional importance of the ruminant large intestine. In sheep, which are small ruminants, even if most fermentation occurs in the rumen, as much as 27% of dietary cellulose could be digested daily in the colon, with the resulting acetate, propionate, and butyrate production accounting for 8% to 17% of the total energy produced daily.^30^ Comparable concentrations of those SCFAs were measured in the rumen, reticulum, and omasum vs. the cecum, colon, and rectum of dairy cows, which are large ruminants.^128^

In non-herbivorous animals, the contribution of SCFAs to the host energy requirement is surprisingly high. In rodents, it varies between 5% and 19% depending on the location in the large intestine and the animal species.^129^ In growing pigs, SCFAs from fiber fermentation can contribute to up to 30% of the energy requirements.^80^ Insoluble dietary fibers are mostly fermented in the pig colon and probably contribute to a significant amount of the energy required by these animals.^130^ As early as 1916, Rubner reported that humans are also able to digest cellulose. He found that 80% of the cellulose in fruits and vegetables disappears, 40% from bread of various millings. These high figures were certainly overestimated because of the lack of accuracy of the biochemical method used at that time. A few recent studies have investigated cell wall digestibility in humans. The daily consumption of plant cell walls is 10–25 g for an adult European, i.e., about 30% of the dietary fiber ingested.^27^ Moreover, the digestibility of cellulose in a group of seven women on a standardized diet was estimated to be 70%. In the same study, only 8% of an added refined cellulose was digested. The type of cellulose appears to be critical.^131^ In humans, more bacteria are able to grow on sources of hydrated, amorphous cellulose, such as spinach cell walls, compared with bacteria that are able to degrade largely crystalline cellulose substrates, such as milled filter paper.^16^ In the large intestine of humans, it is estimated that the chain of degradation of complex carbohydrates from plant materials produces 5%–10% of the human energy requirements.^132^ Acetate is the major energy contributor, accounting for half of the production of total volatile fatty acids in the large intestine ecosystem in omnivorous mammals, such as humans,^133^ and up to three quarters in herbivorous, such as ruminants or horses.^11,129^

## Effects of cellulose-degrading bacteria on health

In addition to providing energy for herbivorous and omnivorous mammals, SCFAs play beneficial roles in the host health and in the microbiota–gut-health communication, that have been recently summarized in two reviews.^134,135^ Among SCFAs, butyrate has been identified has a main contributor of the host intestinal health as it improves the large intestine barrier integrity and function,^136–144^ protects from local intestinal inflammation^145–150^ and stimulates the host local immune reactivity.^151,152^ Cellulolytic bacteria are able to generate large quantities of acetate, succinate and formate from dietary plant cell walls, which may support the production of butyrate by other members of the microbial ecosystem. It has been hypothesized that succinate and acetate produced by *R. champanellensis* can be used in turn by other bacteria for producing propionate and butyrate, respectively.^153^ Propionate producers are *Bacteroidetes* and *Veillonellaceae*, and butyrate producers are *Faecalibacterium prausnitzii, Eubacterium rectale, Roseburia* spp., *Eubacterium hallii*, and *Anaerostipes* spp.^15^ It has been observed that *R. champanellensis* and strains of *Enterococcus* sp. were mainly identified in methane-excreting subjects.^27,68^ This could be explained by the fact that these cellulolytic bacterial strains produce hydrogen which can be used by the methanogenic bacteria.^25,27,68^ The crucial implication of cellulolytic bacteria may explain the fact that high-fiber diets are recognized for their valuable effects on health. For example, in rabbits, a high-fiber diet prevented the digestive troubles, mainly diarrhea, associated with a lower-fiber diet.^65^ Similar results were obtained in the pig, in which fibers prevented diarrhea.^154–156^ SCFAs might influence gut–brain communication and brain function directly or indirectly through immune, endocrine, vagal and other humoral pathways.^135^ To which extent the contribution of each of the major SCFAs produced by cellulolytic bacteria are involved has not been established.

Beyond the influence that cellulolytic bacteria exert via the indirect action of their end products, they also play a direct role in maintaining gut microbial ecosystem and homeostasis. As primary degraders, they play a key role in initiating a network of metabolic interactions that provide a large flow of carbon and energy that may be ‘shared’ with the rest of the microbial community.^15,16,33^ Their absence may have wide-ranging consequences for the whole community.^33^ If cellulose remains largely undegraded, provision of carbon and energy will indeed be decreased for other microorganisms that do not have the ability to access complex polysaccharides, and therefore microbiota diversity and functionality of the ecosystem will be reduced.^157^ Certain commensal microorganisms that normally act as a protective barrier against pathogens may see their abundance decreased in case of low diversity microbiota. This can disrupt the balance between commensal bacterial species and pathogenic species and even lead to infection. The alteration of the microbial ecosystem diversity and functionality can be damaging to the homeostasis of the host considering that nutrition, immunity and metabolism are largely governed by intricate host-microbiota relationships.^158–160^ As an example, exposure to antibiotics is a major cause of gut microbiota disruption in human. This precedes the development of *Clostridioides* (*Clostridium) difficile*, a resident bacteria that can lead to intestinal disease ranging from mild to severe diarrhea and severe complication such as pseudomembranous colitis, toxic megacolon, or even death.^161^ Different commensal bacterial species and strains have been shown to impact *C. difficile* behavior and virulence via interspecies interactions.^162^ Whether cellulolytic bacteria and *C. difficile* interacts is not specifically described yet. However, a study conducted in horses treated with antibiotics, reported that cellulolytic bacteria decreased drastically during the week of treatment and in the following week of withdrawal, while *Salmonella* and *C. difficile* increased in the healthy horse feces.^163^ Additional investigation on how cellulolytic bacteria and their metabolites can modulate the growth of pathogens and further impact host innate and adaptive immune response to these pathogens is needed to understand the development of diseases.

## Conclusion

Since the nineteenth century, the breakdown of dietary cellulose by large intestine bacteria has been the subject of research in both herbivorous and omnivorous mammals, including humans. Numerous studies have been conducted on bacteria whereas fungi^164,165^ and protozoa^166^ have been less investigated. However, it was shown in the rumen by an analysis that targeted mRNA of eukaryotic origin, and resulted in the discovery of a very high number of glycosyl hydrolase genes.^167^ The interest of these microorganisms in the large intestine could also be investigated. The role of cellulolytic bacteria, mostly of the *Fibrobacter* and *Ruminococcus* genera, has been mainly studied because of their keystone place in the metabolic chain and their beneficial effects on host nutrition and gut health, which is attributed to SCFA production. However, very few representatives of cellulolytic bacteria have been isolated and characterized from the large intestine of mammals, mainly because of technical constraints related to their specific growth requirements under strict anaerobic conditions. This results in a lack of knowledge on their physiology, ecology, enzymatic systems, and genome. Describing the genome of new cellulolytic bacteria strains would improve their identification using molecular biology methods. Many studies emphasized that the majority (40%–88%) of bacterial 16S rRNA coding sequences correspond to unidentified strains from various animals^79,168^ and humans.^169,170^ Therefore, it is possible that, within this rate of unknown bacterial species, there may be new cellulolytic bacterial species that have not been cultured and identified. Future studies will serve to inform on the presence of new species and new strains of cellulolytic bacteria, to better understand them for a good functioning in their natural environment. This ultimately will serve to improve plant cell wall utilization or to restore the large intestine homeostasis after dysbiosis occurring under stresses such as diet changes, antibiotic treatment, or microbial infection.

## Data Availability

Data sharing is not applicable to this article as no new data were created or analyzed in this study.
